# Dendritic Cell Protection from Cisplatin Nephrotoxicity Is Independent of Neutrophils

**DOI:** 10.3390/toxins7083245

**Published:** 2015-08-19

**Authors:** Raghu K. Tadagavadi, Guofeng Gao, Wei Wei Wang, Manuel Rovira Gonzalez, W. Brian Reeves

**Affiliations:** Division of Nephrology, the Penn State College of Medicine, 500 University Drive, Hershey, PA 17033, USA; E-Mails: tkraghu@gmail.com (R.T.K.); guofgao@gmail.com (G.G.); www2@psu.edu (W.W.W.); noli_rovira@hotmail.com (M.R.G.)

**Keywords:** cisplatin, kidney injury, nephrotoxicity, dendritic cells, neutrophils

## Abstract

Cisplatin is a very effective chemotherapeutic agent used against a wide range of solid tumors. A major adverse effect of cisplatin therapy is acute kidney injury (AKI). Neutrophils are reported to infiltrate and exacerbate injury in a wide range of sterile inflammatory models of tissue injury. Here, we studied the kinetics of neutrophil infiltration into kidneys and their role in cisplatin-mediated AKI. Mice treated with cisplatin showed an increase in circulating neutrophils 24 and 48 h after cisplatin administration. Cisplatin treatment caused an increase in kidney leukocytes with neutrophils accounting for the majority of the infiltrating leukocytes. The extent of neutrophil infiltration coincided with the severity of kidney injury and renal dysfunction. To examine the functional relevance of infiltrating neutrophils in cisplatin nephrotoxicity, we depleted neutrophils using a neutrophil-specific antibody (anti-Ly-6G). This antibody resulted in greater than 90% depletion of neutrophils in both the blood and kidney. Of note, depletion of neutrophils had no impact on the extent of cisplatin-induced AKI as compared to non-depleted mice. Earlier, we reported that dendritic cell depletion in CD11c-DTRtg mice causes exacerbation of AKI and a dramatic increase in renal neutrophils. Thus, we also examined the role of neutrophils in dendritic cell-depleted mice treated with cisplatin. Dendritic cell depletion exacerbated AKI in spite of neutrophil depletion. These data demonstrate that cisplatin nephrotoxicity is not mediated by neutrophils and that dendritic cells protect kidneys via neutrophil-independent mechanisms.

## 1. Introduction

Cisplatin is a very effective chemotherapeutic agent used for the treatment of a variety of solid tumors. A major toxicity of cisplatin chemotherapy is acute kidney injury. Innate immune responses are pathogenic in both ischemic and toxic acute kidney injury [1,2,3,4]. Dendritic cells represent a major population of immune cells in the kidney [5,6,7]. Dendritic cells are sentinels of the immune system and, depending on the context, can exert anti-inflammatory immunomodulatory actions [8,9] or, in response to pathogens or products of tissue injury, can mature and initiate immunity or inflammatory diseases [10]. In earlier work, we used mice which express the simian diphtheria toxin (DT) receptor driven by the dendritic cell CD11c promoter (CD11c-DTRtg), to investigate the role of dendritic cells in acute cisplatin nephrotoxicity [6]. Those results indicated that dendritic cells reduced cisplatin nephrotoxicity. Subsequent work revealed that the production of the anti-inflammatory cytokine IL-10 by dendritic cells was partially responsible for this protective effect [11].

During cisplatin nephrotoxicity, there is an extensive infiltration of neutrophils into the kidney [1,6,12,13]. We also noted that mice depleted of dendritic cells had markedly increased numbers of neutrophils infiltrating the kidneys [6]. A recent study found that depletion of dendritic cells using diphtheria toxin can itself induce systemic neutrophilia [14]. Together, these observations raised the question of whether the exacerbation of cisplatin nephrotoxicity we observed upon depletion of dendritic cells was due to an increase in neutrophils rather than the absence of dendritic cells. The role of neutrophils in acute kidney injury remains controversial [15,16]. Therefore, the current studies were performed to: 1. Evaluate the role of neutrophils in cisplatin nephrotoxicity and; 2. determine if the effects of dendritic cell depletion in cisplatin nephrotoxicity are neutrophil dependent.

## 2. Results

**Figure 1:**
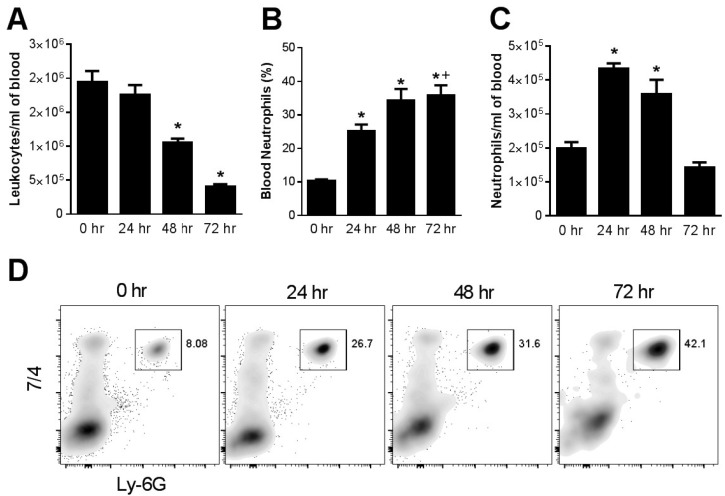
Effect of cisplatin on blood leukocytes and neutrophils. Blood collected from WT mice at different time intervals after cisplatin injection was analyzed for (**A**) total leukocytes (*, *p* < 0.05 *vs*. all other groups), (**B**) neutrophil percentage (*, *p* < 0.01 *vs.* 0 h, +, *p* < 0.05 *vs.* 24 h) and (**C**) absolute neutrophil count (*, *p* < 0.005 *vs*. 0 h and 72 h), *n* = 5 per group. (**D**) flow cytometry.

**Figure 2:**
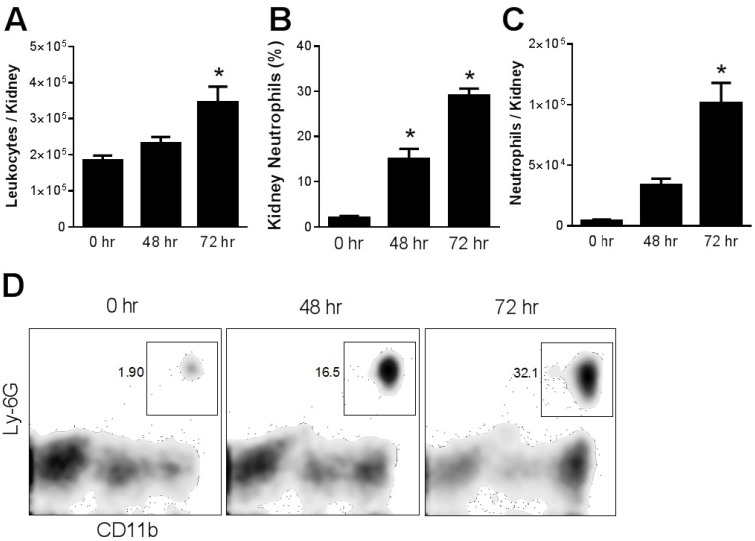
Renal infiltration of leukocytes and neutrophils in cisplatin nephrotoxicity. WT mice administered cisplatin were euthanized at different time intervals and single cell suspensions of kidney were analyzed by flow cytometry for CD45+ leukocytes (**A**) and for neutrophils (**B**–**D**). *, *p* < 0.02 *vs*. each other group*. n* = 5.

Effect of cisplatin on circulating and kidney-infiltrating neutrophils. Blood and kidney tissue were subjected to flow cytometry analysis before and at 24 h intervals following the administration of a single, nephrotoxic, dose of cisplatin. The total leukocyte count in the peripheral blood decreased over the 72 h after cisplatin administration. The percentage of neutrophils in blood increased 2–3 fold and persisted at 72 h. As a result, the absolute number of circulating neutrophils was increased at 24 and 48 h before falling at 72 h (Figure 1). Even more dramatic changes were observed in the kidney, where there was a progressive increase in total leukocytes over the 72 h following cisplatin injection. Neutrophils were a prominent component of this inflammatory infiltrate and accounted for 30% of total kidney leukocytes compared to less than 5% in untreated mice (Figure 2).

**Figure 3:**
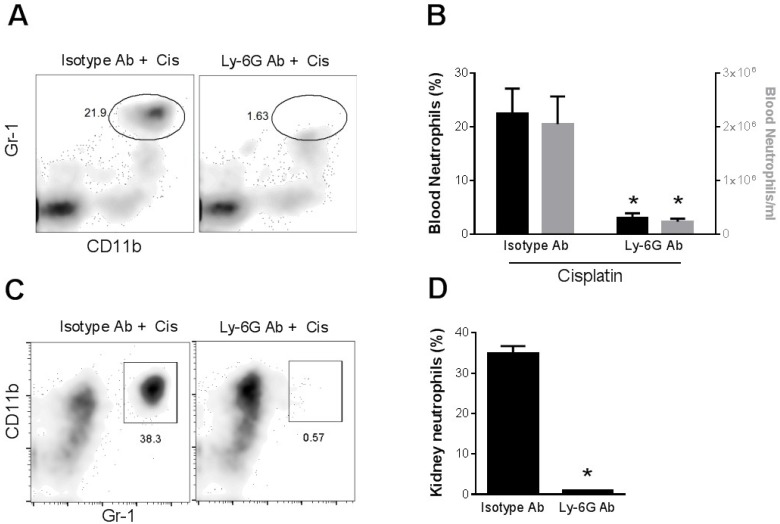
Depletion of neutrophils in blood and kidney of cisplatin-treated mice with Ly-6G antibody. (**A**) Mice were administered an isotype antibody or Ly-6G antibody 6 h before cisplatin injection. Blood was collected 24 h after cisplatin treatment and analyzed for circulating neutrophils by flow cytometry. (**B**) Summary results. (**C**) Mice were administered an isotype antibody or Ly-6G antibody 24 h before and 24 h after cisplatin injection. Kidneys were harvested 72 h after cisplatin treatment and single cell suspensions were analyzed by flow cytometry after gating on the CD45 leukocyte marker for neutrophils. (**D**) Summary results. * *p* < 0.02 *versus* isotype antibody*. n* = 4.

**Figure 4:**
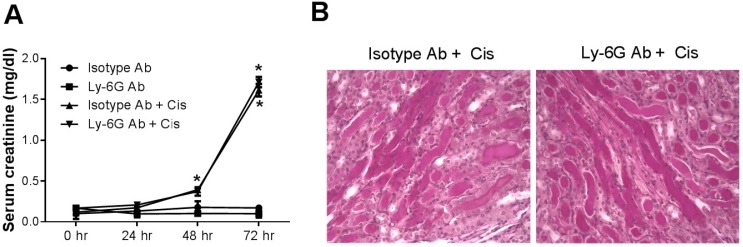
Effect of neutrophil depletion on cisplatin nephrotoxicity. (**A**) Kidney function as measured by serum creatinine in mice treated with cisplatin or saline and either isotype antibody or Ly-6G antibody. (*n* = 3–5). *, *p* < 0.01 *vs.* antibody without cisplatin. (**B**) Photomicrographs of PAS-stained kidney sections (40X) harvested 72 h after cisplatin injection to mice treated with either isotype control antibody (**left**) or Ly-6G antibody (**right**).

Role of neutrophils in cisplatin nephrotoxicity. In order to assess the role of neutrophils in cisplatin nephrotoxicity, we injected mice with an antibody (1A8) against Ly-6G, a neutrophil marker. This antibody has been shown to produce more specific deletion of neutrophils [17,18] than the Gr1 antibody (RB6-8C5) used in some prior studies [19,20]. Figure 3 shows that 1A8, but not an isotype control antibody, resulted in virtually complete depletion of circulating neutrophils in cisplatin-treated mice. Likewise, 1A8 effectively depleted neutrophils within the kidney of cisplatin-treated mice (Figure 3C,D). In spite of the depletion of neutrophils in both the circulation and the kidney, kidney function, as judged by serum creatinine levels (Figure 4A) or blood urea nitrogen levels (data not shown), was decreased by cisplatin to a similar extent in both groups of mice Histologic injury, as shown by tubular dilation, loss of brush border membranes, cast formation and sloughing of dead or injured renal epithelial cells, was also similar in the neutrophil-depleted mice compared with non-depleted mice (Figure 4).

**Figure 5:**
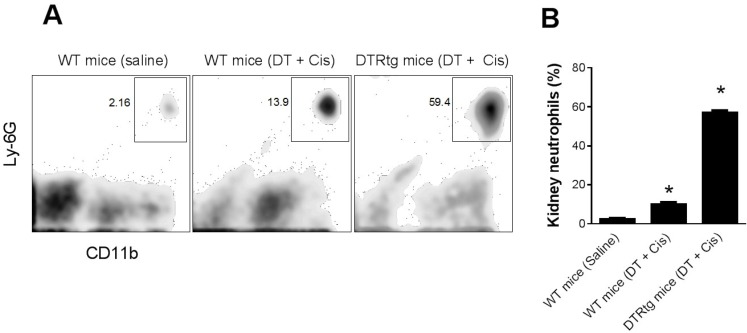
Dendritic cell ablation augments kidney neutrophil infiltration in cisplatin nephrotoxicity. WT and CD11c-DTRtg mice administered saline or DT and cisplatin were sacrificed 24 h after cisplatin. (**A**) Single cell suspensions of kidneys were analyzed by flow cytometry for neutrophils. (**B**) Summary results. *, *p* < 0.01 *versus* each other group*. n* = 3–5.

**Figure 6:**
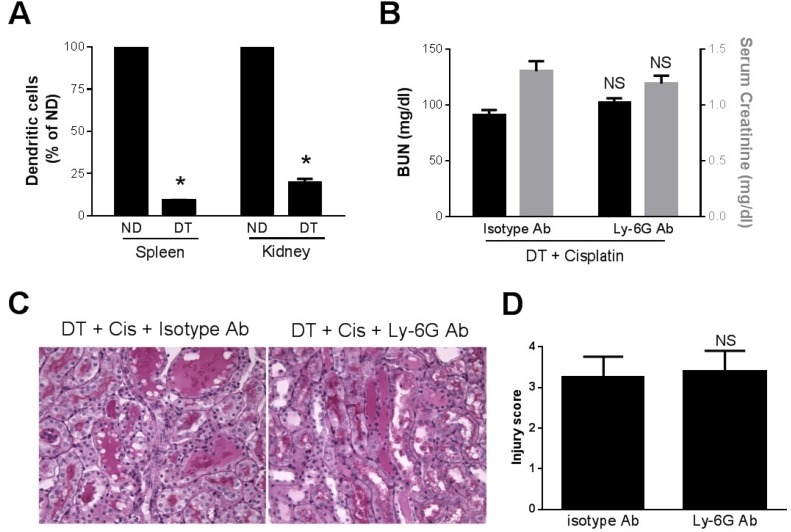
Effect of dendritic cell depletion and neutrophil depletion on cisplatin nephrotoxicity. CD11c-DTR mice were treated with DT and cisplatin and either isotype antibody or Ly-6G antibody. (**A**) Dendritic cells in spleen and kidney were counted using FACS analysis in mice which had (DT) or had not (ND) been treated with DT. *, *p* < 0.05 *vs.* ND (**B**) Blood was collected 24 h after cisplatin and BUN (black bars) and creatinine (gray bars) were measured as indices of kidney function. (**C**) Photomicrographs of PAS-stained kidney sections (40×) harvested 24 h after cisplatin injection to mice treated with DT and either isotype control antibody (**left**) or Ly-6G antibody (**right**). (**D**) Tubular injury scores. NS—not significant *vs.* isotype Ab, *n =* 3–6*.*

Role of neutrophils in dendritic cell-mediated kidney protection. Since we found in a previous study that DT-induced depletion of dendritic cells resulted in increased kidney neutrophils and cisplatin kidney injury [6] and Tittel *et al.* [14] reported increased neutrophilia after DT-induced dendritic cell depletion, we sought to determine if the previously observed effects of DT were related to neutrophilia. First, we analyzed the accumulation of neutrophils in the kidney in CD11c-DTRtg mice treated with DT to deplete dendritic cells followed by cisplatin. At 24 h after cisplatin, WT mice exhibited a small increase in kidney neutrophils. However, this was strikingly increased in mice which had been depleted of dendritic cells (Figure 5). Next, we examined kidney injury in the DC-depleted mice. Shown in Figure 6A are the serum creatinine levels obtained 24 h after cisplatin injection. As we reported previously [6], the depletion of dendritic cells was associated with significantly more severe, and more rapid, kidney dysfunction. The creatinine level at 24 h was markedly elevated in DC-depleted mice. In contrast, the serum creatinine remains near normal 24 h after cisplatin in WT mice (see Figure 4A). To determine if the worsening of kidney failure in the DC-depleted mice was due to the increased neutrophil infiltration, we treated CD11c-DTRtg mice with DT and either 1A8 or isotype control antibody followed by cisplatin. The early onset and increased extent of kidney failure was evident in the dendritic cell-depleted mice and there was no significant difference in either the BUN or creatinine levels regardless of whether neutrophils were intact or depleted (Figure 6). Histology demonstrated similar degrees of tubular injury in both groups of mice (Figure 6C,D).

## 3. Discussion

Neutrophils are a prominent component of the sterile inflammatory response seen after ischemic or toxic injury to the kidney or other organs. In response to tissue injury, damage-associated molecular pattern molecules (DAMPs), such as high mobility group box 1 (HMGB1), are released from injured cells. These DAMPs bind to TLR receptors on endothelial cells and immune cells and activate innate immune responses including the production of chemokines and cytokines and upregulation of leukocyte adhesion molecules [21]. The trafficking of neutrophils after kidney injury has been studied after ischemia-reperfusion [22,23]. In that setting, neutrophils begin to adhere to the endothelium within 2 h of ischemia and subsequently transmigrate into the tubulointerstitium [22]. Neutrophils release a variety of substances, such as reactive oxygen species, proteases and cytokines which could potentially result in injury to the surrounding kidney parenchymal cells [15]. Nonetheless, the role of neutrophils in acute kidney injury continues to be controversial [15,21]. On the one hand, maneuvers which impair the recruitment of neutrophils, such as inhibition or deletion of adhesion molecules [24,25,26] or chemokines [20], have been shown to reduce ischemic and toxic AKI. However, depletion of neutrophils using antibodies has yielded variable results, with recent studies showing no effect of neutrophil depletion on kidney function after ischemia [16,27].

Less is known regarding the pathogenic role of neutrophils in cisplatin nephrotoxicity. Toxic kidney injury in response to agents such as cisplatin [1,6,19] or mercury [28], results in the neutrophil infiltration in the kidney. In the present report, we confirmed that neutrophils progressively infiltrate into the kidney after cisplatin treatment. We also found, like Faubel *et al.* [19], that cisplatin treatment resulted in peripheral neutrophilia. Two prior studies used neutrophil-depleting antibodies to explore their pathogenic role in a murine model of cisplatin nephrotoxicity [19,29]. The first study used an antibody directed against GR-1 (RB6-8C5) and found no effect no nephrotoxicity [19]. The GR-1 antibody, however, binds to both Ly6G and Ly6C and is not specific for neutrophils [17,18] and may have depleted monocytes which could have obscured the effects of neutrophil depletion. The second study used a neutrophil specific antibody (anti-Ly-6G) and found a modest, but not statistically significant, reduction in nephrotoxicity [29]. We used the same antibody as in the latter study and observed no improvement in kidney function even though neutrophil depletion was virtually complete. Taken together, these results suggest that in spite of high levels of neutrophils in the kidney and circulation, neutrophils play a small role, if any, in the pathogenesis of cisplatin nephrotoxicity. Likewise, in drug-induced liver injury, which is also characterized by neutrophil infiltration, depletion of neutrophils does not reduce tissue injury [30,31]. This study, and others [19,29], have only examined the initial injury after acute cisplatin administration. The possibility that neutrophils might play either a beneficial or harmful role in the recovery from acute nephrotoxicity remains to be determined. We note that mice have a lower percentage of circulating neutrophils than humans [32]. However, the percentage of neutrophils in mice increased after cisplatin treatment to a range often observed in humans (Figure 1B), suggesting that our results in mice may be relevant to humans.

We have recently shown that dendritic cells, which are the most abundant immune cell in the normal kidney [6,33], are protective against cisplatin nephrotoxicity [6]. This conclusion was derived from studies in the CD11c-DTRtg mouse in which expression of the simian diptheria toxin receptor driven by the CD11c promoter targets dendritic cells for DT-mediated cell death [10]. That model has been criticized based on recent findings that DT-mediated depletion results in systemic neutrophilia which may produce effects independent of dendritic cell depletion [14]. Indeed, we had observed that DT-treated mice displayed much higher rates of kidney neutrophil infiltration after cisplatin treatment than did non-DT treated mice [6]. These findings raised the possibility that the exacerbation we noted in DC-depleted mice could have been a result of either systemic or kidney neutrophilia. Therefore, we assessed the effects of dendritic cell depletion in the absence of neutrophils. Our results confirm our earlier finding [6,11] that dendritic cell depletion results in a marked worsening of cisplatin nephrotoxicity. In addition, they indicate clearly that the exacerbation of kidney injury was independent of neutrophils. The mechanisms whereby dendritic cells protect against cisplatin nephrotoxicity are still under investigation, but include the production of IL-10 [11]. Although cisplatin nephrotoxicity was found to be independent of neutrophils, dendritic cells could provide protection through other leukocyte populations. In this regard, immature dendritic cells can induce the production and proliferation of Treg cells, which are known to reduce kidney injury after ischemia-reperfusion [34,35] and cisplatin treatment [36]. In addition, cisplatin may directly reduce the numbers of Treg cells [37]. Dendritic cells could also modulate kidney injury by altering the Th1/Th2 polarization of effector T cells [38]. Mast cells have also been implicated in cisplatin toxicity as a source of TNF [39]. Although mast cell TNF can stimulate dendritic cells to mature [40], the effects of dendritic cells on mast cell function is poorly understood.

In summary, cisplatin results in an increase in circulating and kidney neutrophils in concert with a decrease in kidney function. However, depletion of neutrophils has no beneficial impact on either functional or structural kidney damage after cisplatin. In addition, although the depletion of dendritic cells worsens both cisplatin nephrotoxicity and kidney neutrophil infiltration, the effects on nephrotoxicity are independent of neutrophils. These results suggest that neutrophils have little role in acute cisplatin kidney injury. Further studies will be needed to assess the role of neutrophils in chronic cisplatin toxicity or in the recovery after acute kidney injury.

## 4. Experimental Section

Mice. Experiments were performed using 8- to 10- week old C57BL6 mice and CD11c-DTRtg mice (B6.FVB-Tg Itgax-DTR/GFP 57Lan/J) harboring a transgene encoding a simian diphtheria toxin receptor/green fluorescent protein (DTR/GFP) fusion protein under the transcriptional control of mouse CD11c promoter. Acute kidney injury was induced in mice by injecting a single dose of cisplatin (20 mg/kg body weight), intraperitoneally [1]. To deplete neutrophils, mice were injected intraperitoneally with 400 µg of an Ly-6G antibody (clone 1A8, Bio X Cell, West Lebanon, NH, USA) or an isotype control antibody 6 h prior to cisplatin injection and again 24 h after cisplatin injection. Dendritic cells were ablated in CD11c-DTR mice by intraperitoneal injection of DT (4 ng/gm body weight) 24 h before cisplatin injection [6,11]. Animals were used according to protocols approved by the IACUC of The Pennsylvania State University College of Medicine.

Kidney injury markers. Kidney function was determined by measuring BUN (VITROS DT60II chemistry slides; Ortho-Clinical Diagnostics, Rochester, NY, USA) and serum creatinine (DZ072B; Diazyme labs, Poway, CA, USA). Kidneys were fixed in buffered formalin for 24 h, embedded in paraffin, sectioned (4 µm thickness), and stained with periodic acid-Schiff. The extent of tubular injury was assessed using a semiquantitative scale as described previously [1].

Flow cytometry. Blood was treated with 2% acetic acid solution in distilled water to determine total leukocyte count using Neubauer hemacytometer. Kidneys harvested at sacrifice were processed into single-cell suspensions for flow cytometry as described before (15). Briefly, kidneys were minced into fragments of 1 mm^3^ and digested with 2 mg/mL of collagenase D and 100 U/mL of DNase I for 45 min. The digested kidneys were passed through 100 µm followed by 40 µm mesh. Red blood cells in the resulting renal suspension were lysed using red blood cell lysis buffer (Sigma-Aldrich, St. Louis, MO, USA).

Kidney or blood cells were treated with rat anti-FcR from 2.4G2 hybridoma supernatant to block Fc receptors, and then stained using the following fluorochrome-labeled antibodies; anti-CD45 (clone 30-F11), CD11b (M1/70, eBioscience, San Diego, CA, USA), 7/4 (AbD Serotech, Raleigh, NC, USA) and Ly-6G (1A8, BioLegend, San Diego, CA, USA). Blood after immunofluorescence staining was treated with fixation and permeabilization buffer (BD Biosciences, San Jose, CA, USA) for lysing of red blood cells and fixing of leukocytes. Flow cytometry was performed on BD FACSCalibur or, BD LSR II flow cytometer and analyzed using FlowJo software.

Statistical analysis. Results were expressed as mean ± SE. Data were analyzed using unpaired two-tailed *t* test or one-way ANOVA with Sidek’s multiple comparison test if more than two groups were compared using Prism (v6, GraphPad Software, La Jolla, CA, USA). A value of *p* < 0.05 was considered significant.

## References

[1] Ramesh G., Reeves W.B. (2002). Tnf-a mediates chemokine and cytokine expression and renal injury in cisplatin nephrotoxicity. J. Clin. Investig..

[2] Zhang B., Ramesh G., Uematsu S., Akira S., Reeves W.B. (2008). Tlr4 signaling mediates inflammation and tissue injury in nephrotoxicity. J. Am. Soc. Nephrol..

[3] Li L., Huang L., Sung S.S.J., Lobo P.I., Brown M.G., Gregg R.K., Engelhard V.H., Okusa M.D. (2007). Nkt cell activation mediates neutrophil ifn-{gamma} production and renal ischemia-reperfusion injury. J. Immunol..

[4] Li L., Huang L., Vergis A.L., Ye H., Bajwa A., Narayan V., Strieter R.M., Rosin D.L., Okusa M.D. (2010). Il-17 produced by neutrophils regulates ifn-g mediated neutrophil migration in mouse kidney ischemia-reperfusion injury. J. Clin. Investig..

[5] Bamboat Z.M., Ocuin L.M., Balachandran V.P., Obaid H., Plitas G., DeMatteo R.P. (2010). Conventional dcs reduce liver ischemia/reperfusion injury in mice via il-10 secretion. J. Clin. Investig..

[6] Tadagavadi R.K., Reeves W.B. (2010). Renal dendritic cells ameliorate nephrotoxic acute kidney injury. J. Am. Soc. Nephrol..

[7] Kruger T., Benke D., Eitner F., Lang A., Wirtz M., Hamilton-Williams E.E., Engel D., Giese B., Muller-Newen G., Floege J. (2004). Identification and functional characterization of dendritic cells in the healthy murine kidney and in experimental glomerulonephritis. J. Am. Soc. Nephrol..

[8] Munn D.H., Sharma M.D., Lee J.R., Jhaver K.G., Johnson T.S., Keskin D.B., Marshall B., Chandler P., Antonia S.J., Burgess R. (2002). Potential regulatory function of human dendritic cells expressing indoleamine 2,3-dioxygenase. Science.

[9] Laouar Y., Town T., Jeng D., Tran E., Wan Y., Kuchroo V.K., Flavell R.A. (2008). Tgf-beta signaling in dendritic cells is a prerequisite for the control of autoimmune encephalomyelitis. Proc. Natl. Acad. Sci. USA.

[10] Jung S., Unutmaz D., Wong P., Sano G., De los Santos K., Sparwasser T., Wu S., Vuthoori S., Ko K., Zavala F. (2002). *In vivo* depletion of cd11c+ dendritic cells abrogates priming of CD8+ T cells by exogenous cell-associated antigens. Immunity.

[11] Tadagavadi R.K., Reeves W.B. (2010). Endogenous il-10 attenuates cisplatin nephrotoxicity: Role of dendritic cells. J. Immunol..

[12] Ramesh G., Reeves W.B. (2003). Tnfr2-mediated apoptosis and necrosis in cisplatin-induced acute renal failure. Am. J. Physiol Ren. Physiol..

[13] Zhang B., Ramesh G., Norbury C., Reeves W.B. (2007). Cisplatin-induced nephrotoxicity is mediated by tumor necrosis factor-a produced by renal parenchymal cells. Kidney Int..

[14] Tittel A.P., Heuser C., Ohliger C., Llanto C., Yona S., Hammerling G.J., Engel D.R., Garbi N., Kurts C. (2012). Functionally relevant neutrophilia in CD11c diphtheria toxin receptor transgenic mice. Nat. Methods.

[15] Bolisetty S., Agarwal A. (2009). Neutrophils in acute kidney injury: Not neutral any more. Kidney Int..

[16] Kumar S., Allen D.A., Kieswich J.E., Patel N.S., Harwood S., Mazzon E., Cuzzocrea S., Raftery M.J., Thiemermann C., Yaqoob M.M. (2009). Dexamethasone ameliorates renal ischemia-reperfusion injury. J. Am. Soc. Nephrol..

[17] Daley J.M., Thomay A.A., Connolly M.D., Reichner J.S., Albina J.E. (2008). Use of ly6g-specific monoclonal antibody to deplete neutrophils in mice. J. Leukoc. Biol..

[18] Carr K.D., Sieve A.N., Indramohan M., Break T.J., Lee S., Berg R.E. (2011). Specific depletion reveals a novel role for neutrophil-mediated protection in the liver during listeria monocytogenes infection. Eur. J. Immunol..

[19] Faubel S., Lewis E.C., Reznikov L., Ljubanovic D., Hoke T.S., Somerset H., Oh D.J., Lu L., Klein C.L., Dinarello C.A. (2007). Cisplatin-induced acute renal failure is associated with an increase in the cytokines interleukin (il)-1beta, il-18, il-6, and neutrophil infiltration in the kidney. J. Pharmacol. Exp. Ther..

[20] Miura M., Fu X., Zhang Q.W., Remick D.G., Fairchild R.L. (2001). Neutralization of gro alpha and macrophage inflammatory protein-2 attenuates renal ischemia/reperfusion injury. Am. J. Pathol..

[21] Jang H.R., Rabb H. (2009). The innate immune response in ischemic acute kidney injury. Clin. Immunol..

[22] Awad A.S., Rouse M., Huang L., Vergis A.L., Reutershan J., Cathro H.P., Linden J., Okusa M.D. (2009). Compartmentalization of neutrophils in the kidney and lung following acute ischemic kidney injury. Kidney Int..

[23] Ysebaert D.K., De Greef K.E., Vercauteren S.R., Ghielli M., Verpooten G.A., Eyskens E.J., De Broe M.E. (2000). Identification and kinetics of leukocytes after severe ischemia/reperfusion renal injury. Nephrol. Dial. Transplant..

[24] Singbartl K.A.I., Forlow S.B., Ley K. (2001). Platelet, but not endothelial, p-selectin is critical for neutrophil-mediated acute postischemic renal failure. FASEB J..

[25] Kelly K.J., Williams W.W., Colvin R.B., Bonventre J.V. (1994). Antibody to intercellular adhesion molecule-1 protects the kidney against ischemic injury. Proc. Natl. Acad. Sci. USA.

[26] Kelly K.J., Meehan S.M., Colvin R.B., Williams W.W., Bonventre J.V. (1999). Protection from toxicant-mediated renal injury in the rat with anti-cd54 antibody. Kidney Int..

[27] Melnikov V.Y., Faubel S., Siegmund B., Lucia M.S., Ljubanovic D., Edelstein C.L. (2002). Neutrophil-independent mechanisms of caspase-1- and il-18-mediated ischemic acute tubular necrosis in mice. J. Clin. Investig..

[28] Nechemia-Arbely Y., Barkan D., Pizov G., Shriki A., Rose-John S., Galun E., Axelrod J.H. (2008). Il-6/il-6r axis plays a critical role in acute kidney injury. J. Am. Soc. Nephrol..

[29] Chan A.J., Alikhan M.A., Odobasic D., Gan P.Y., Khouri M.B., Steinmetz O.M., Mansell A.S., Kitching A.R., Holdsworth S.R., Summers S.A. (2014). Innate il-17a-producing leukocytes promote acute kidney injury via inflammasome and toll-like receptor activation. Am. J. Pathol..

[30] Williams C.D., Bajt M.L., Sharpe M.R., McGill M.R., Farhood A., Jaeschke H. (2014). Neutrophil activation during acetaminophen hepatotoxicity and repair in mice and humans. Toxicol. Appl. Pharmacol..

[31] Williams C.D., Bajt M.L., Farhood A., Jaeschke H. (2010). Acetaminophen-induced hepatic neutrophil accumulation and inflammatory liver injury in cd18-deficient mice. Liver Int..

[32] Haley P.J. (2003). Species differences in the structure and function of the immune system. Toxicology.

[33] Soos T.J., Sims T.N., Barisoni L., Lin K., Littman D.R., Dustin M.L., Nelson P.J. (2006). Cx3cr1+ interstitial dendritic cells form a contiguous network throughout the entire kidney. Kidney Int..

[34] Gandolfo M.T., Jang H.R., Bagnasco S., Ko G.J., Agreda P., Satpute S., Crow M.T., King L.S., Rabb H. (2009). Foxp3+ regulatory t cells participate in repair of ischemic acute kidney injury. Kidney Int..

[35] Kinsey G.R., Sharma R., Huang L., Li L., Vergis A.L., Ye H., Ju S.T., Okusa M.D. (2009). Regulatory t cells suppress innate immunity in kidney ischemia-reperfusion injury. J. Am. Soc. Nephrol..

[36] Lee H., Nho D., Chung H.S., Lee H., Shin M.K., Kim S.H., Bae H. (2010). CD4+CD25+ regulatory t cells attenuate cisplatin-induced nephrotoxicity in mice. Kidney Int..

[37] Huang X., Chen Y.T., Song H.Z., Huang G.C., Chen L.B. (2011). Cisplatin pretreatment enhances anti-tumor activity of cytokine-induced killer cells. World J. Gastroenterol..

[38] Maddur M.S., Sharma M., Hegde P., Stephen-Victor E., Pulendran B., Kaveri S.V., Bayry J. (2014). Human B cells induce dendritic cell maturation and favour th2 polarization by inducing ox-40 ligand. Nat. Commun..

[39] Summers S.A., Chan J., Gan P.Y., Dewage L., Nozaki Y., Steinmetz O.M., Nikolic-Paterson D.J., Kitching A.R., Holdsworth S.R. (2011). Mast cells mediate acute kidney injury through the production of TNF. J. Am. Soc. Nephrol..

[40] Suto H., Nakae S., Kakurai M., Sedgwick J.D., Tsai M., Galli S.J. (2006). Mast cell-associated tnf promotes dendritic cell migration. J. Immunol..

